# Chronic Δ^9^-THC Exposure Differently Affects Histone Modifications in the Adolescent and Adult Rat Brain

**DOI:** 10.3390/ijms18102094

**Published:** 2017-10-04

**Authors:** Pamela Prini, Federica Penna, Emanuele Sciuccati, Tiziana Alberio, Tiziana Rubino

**Affiliations:** 1 Department of Biotechnology and Life Sciences, University of Insubria, 21052 Busto Arsizio, VA, Italy; federica.penna@uninsubria.it (F.P.); e.sciuccati@studenti.uninsubria.it (E.S.); tiziana.rubino@uninsubria.it (T.R.); 2 Neuroscience Center, University of Insubria, 21052 Busto Arsizio, VA, Italy; tiziana.alberio@uninsubria.it; 3 Department of Science and High Technology, University of Insubria, 21052 Busto Arsizio, VA, Italy

**Keywords:** Δ^9^-THC, histone modifications, adolescents, adults, nucleus accumbens, amygdala, hippocampus

## Abstract

Adolescence represents a vulnerable period for the psychiatric consequences of delta9-tetrahydrocannabinol (Δ^9^-THC) exposure, however, the molecular underpinnings of this vulnerability remain to be established. Histone modifications are emerging as important epigenetic mechanisms involved in the etiopathogenesis of psychiatric diseases, thus, we investigated the impact of chronic Δ^9^-THC exposure on histone modifications in different brain areas of female rats. We checked histone modifications associated to both transcriptional repression (H3K9 di- and tri-methylation, H3K27 tri-methylation) and activation (H3K9 and H3K14 acetylation) after adolescent and adult chronic Δ^9^-THC exposure in the hippocampus, nucleus accumbens, and amygdala. Chronic exposure to increasing doses of Δ^9^-THC for 11 days affected histone modifications in a region- and age-specific manner. The primary effect in the adolescent brain was represented by changes leading to transcriptional repression, whereas the one observed after adult treatment led to transcriptional activation. Moreover, only in the adolescent brain, the primary effect was followed by a homeostatic response to counterbalance the Δ^9^-THC-induced repressive effect, except in the amygdala. The presence of a more complex response in the adolescent brain may be part of the mechanisms that make the adolescent brain vulnerable to Δ^9^-THC adverse effects.

## 1. Introduction

Despite the accumulation of evidence of a possible relationship between adolescent cannabis abuse and the later development of psychiatric disorders [1,2,3], cannabis remains the most common illegal drug used by adolescents [1]. The high prevalence of its use predicts an increasing amount of young people suffering from psychiatric disorders related to cannabis exposure in the near future. Therefore, there is an urgent need to elucidate the molecular underpinnings that link adolescent cannabis consumption to the development of mental illnesses later in life.

During adolescence the brain undergoes intense structural remodeling [2,3,4], and interfering with these key processes may affect brain functionality and behavior [5]. Adolescence is also the period in which drug consumption begins, and it is known that chronic consumption of drugs of abuse induces important changes in the brain, mainly in the mesocorticolimbic circuitry [6,7].

Recent studies have demonstrated that epigenetic processes may represent the potential mechanism for the translation of environmental stimuli—including drug abuse—into changes of gene expression [8,9]. They mainly include histone modifications, DNA methylation, and small-RNA molecules [10]. Recently, it has been demonstrated that specific histone modifications are involved in gene expression alterations observed after exposure to drugs of abuse [11]. Histone modifications include many types of posttranslational modifications, among which the most studied are acetylation and methylation. Histone acetylation is associated with transcriptional activation, whereas histone methylation can be associated with both transcriptional activation and repression, depending on the site of methylation. For example, on histone H3, methylation of K27 and K9 is associated with repression, whereas methylation on K4 promotes transcriptional activation. These changes might contribute, at least in part, to the etiopathogenesis of drug-induced psychiatric disorders [12].

It is, therefore, possible that cannabis abuse during adolescence could impair the brain network functionality acting through a mechanism involving histone modifications, thus leading to long-term behavioral impairments. Consistent with this hypothesis, we and others have demonstrated that adolescent exposure to delta9-tetrahydrocannabinol (Δ^9^-THC), the psychoactive compound of cannabis, or to synthetic cannabinoids, induced sex-dependent brain and behavioral alterations at adulthood [13,14,15,16,17,18,19,20,21,22,23]. In female rats, the phenotype was more complex, as both depressive-like and psychotic-like signs were present (for more details on sex-dependent vulnerability to cannabis abuse in adolescence see [24] or [25]), thus we decided to further our understanding of adolescent cannabis exposure in females. In the search for a possible mechanism, we demonstrated in the prefrontal cortex (PFC) that Δ^9^-THC alters histone modifications, mainly methylation of H3K9, and the expression of a subset of plasticity genes relevant for the development of cognitive deficits present in the adult phenotype [26]. Interestingly, the alterations induced by Δ^9^-THC exposure were age-specific. Indeed, not only the behavioral phenotype developed after adolescent, and not adult, exposure [16], but also changes in both histone modifications and gene expression were more widespread and intense after adolescent treatment, further confirming a specific adolescent susceptibility [26].

**Figure 1:**
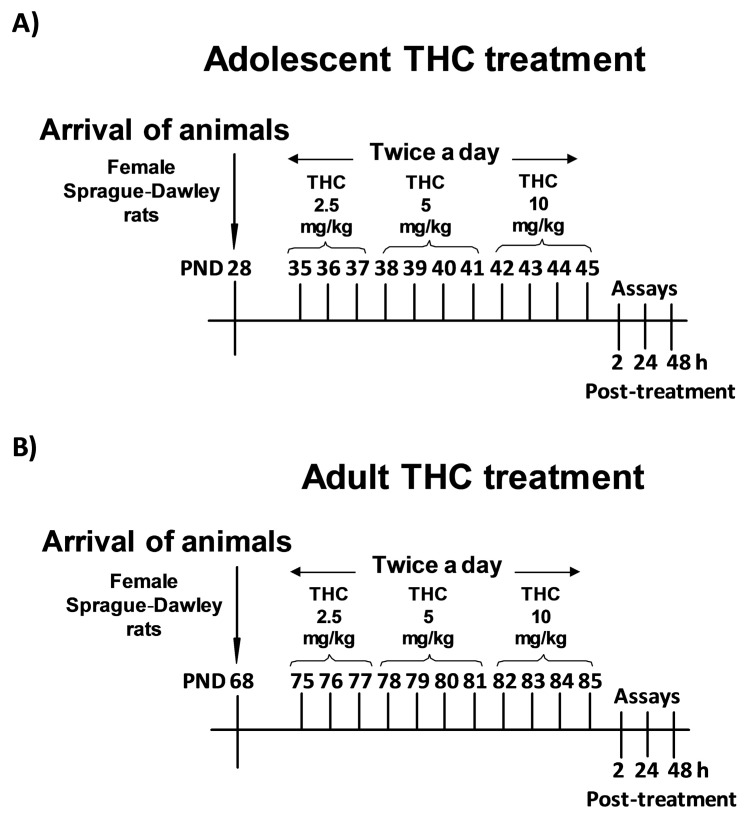
Adolescent (**A**) and adult (**B**) delta9-tetrahydrocannabinol (Δ^9^-THC) treatment schedule.

On these bases, the main goal of this work is to extend our knowledge of the impact of Δ^9^-THC exposure on histone modifications occurring in other brain areas that, together with the PFC, are important for the different aspects of the depressive/psychotic-like phenotype described in these animals [13,14,15,16,17,18]. To this aim, we investigated histone modifications associated with both transcriptional repression (H3K9 di- and tri-methylation, H3K27 trimethylation) and activation (H3K9 and H3K14 acetylation), since they have already been reported to be modulated by drug treatment [10]. These analyses were performed 2, 24, and 48 h after the last Δ^9^-THC injection in the hippocampus (Hippo), nucleus accumbens (NAc), and amygdala (Amy) brain areas, which undergo dramatic changes during adolescence and which are mainly affected by drug consumption [6,7]. To investigate the existence of age-specificity of Δ^9^-THC effects, the study was performed after both adolescent and adult exposure, which consisted of increasing doses of Δ^9^-THC for 11 days, as reported in Figure 1A,B.

## 2. Results

### 2.1. Effect of Adolescent Delta9-Tetrahydrocannabinol (Δ^9^-THC) Exposure on Histone Modifications

**Figure 2:**
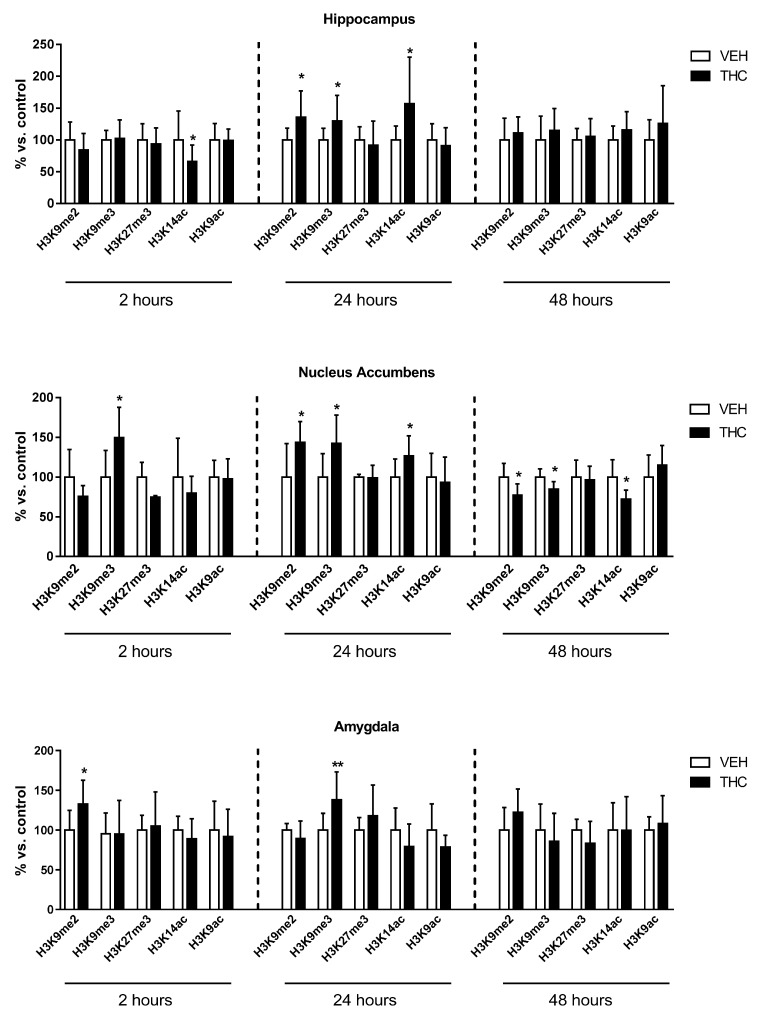
Effect of adolescent Δ^9^-THC exposure on histone modifications occurring in the hippocampus, nucleus accumbens, and amygdala of female rats 2, 24, and 48 h after the last delta9-tetrahydrocannabinol (Δ^9^-THC) or vehicle (VEH) injection. Data are expressed as mean ± Standard Deviation (SD) of six/ten animals for each experimental group. * *p* < 0.05, ** *p* < 0.01 versus controls (Student’s *t*-test analysis).

To investigate the impact of adolescent Δ^9^-THC exposure on histone modifications, we analyzed histone H3 modifications associated with transcriptional repression: di- and tri-methylation of lysine 9 (H3K9me2 and H3K9me3), and tri-methylation of lysine 27 (H3K27me3), as well as histone markers associated with transcriptional activation: acetylation of lysine 9 (H3K9ac), and lysine 14 (H3K14ac). Analyses were performed 2, 24, and 48 h after the last Δ^9^-THC injection in the hippocampus, nucleus accumbens, and amygdala of female adolescent rats (Figure 2).

In the hippocampus, H3K14ac was significantly reduced (34%) 2 h after the last Δ^9^-THC injection, whilst it was significantly enhanced (57%) 24 h later (representative blots are shown in Figure S1). At this same interval of time, a significant increase of H3K9me2 (36%) and H3K9me3 (30%) was also observed. Forty-eight hours after the end of the treatment, all these alterations returned to control values.

In the nucleus accumbens, H3K9me3 was significantly increased 2 h (50%) and 24 h (43%) after the cease of treatment. H3K9me2 (42%) and H3K14ac (26%) levels were also significantly enhanced at 24 h. On the contrary, at the later time point (48 h), all these modifications were significantly reduced (16%, 23%, and 28%, respectively).

In the amygdala, H3K9me2 levels were significantly enhanced (33%) 2 h after the treatment. Twenty-four hours later, while this alteration returned to control, H3K9me3 levels significantly increased (33%). However, no changes were observed at 48 h.

As a whole, these data suggest that adolescent Δ^9^-THC exposure affects histone modifications differently, depending on the brain region under consideration.

### 2.2. Effect of Adult Δ^9^-THC Exposure on Histone Modifications

**Figure 3:**
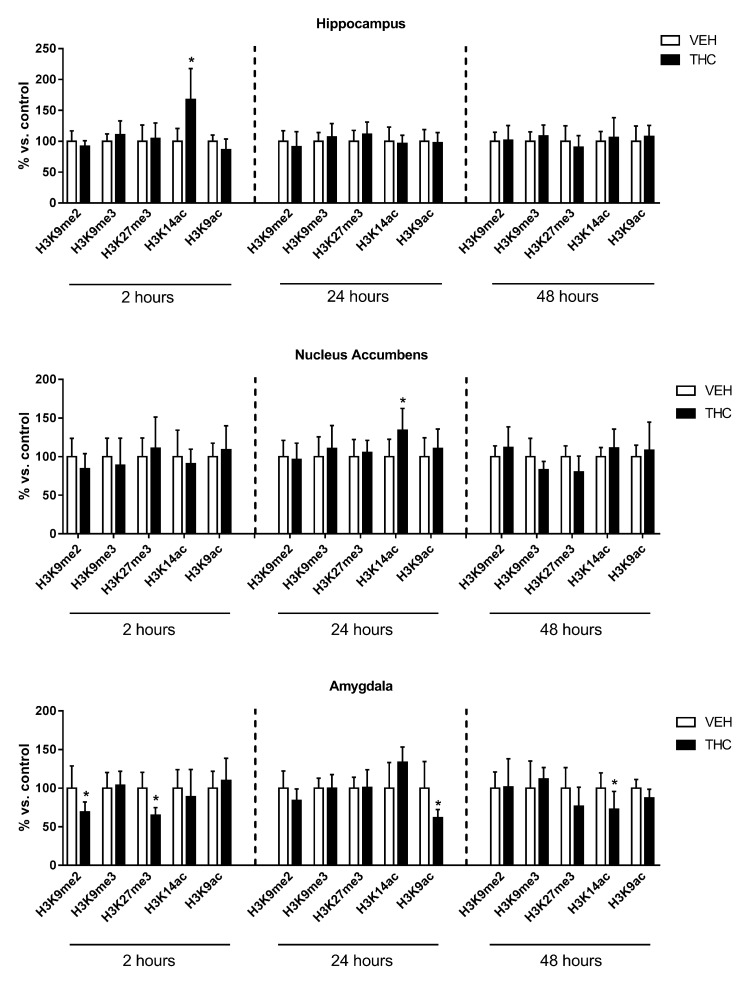
Effect of adult Δ^9^-THC exposure on histone modifications occurring in the hippocampus, nucleus accumbens, and amygdala of female rats 2, 24, and 48 h after the last Δ^9^-THC or vehicle (VEH) injection. Data are expressed as mean ± SD of at least five animals for each experimental group. * *p* < 0.05 versus controls (Student’s *t*-test analysis).

Since the development of the depressive/psychotic-like phenotype is restricted to adolescent Δ^9^-THC exposure [13,14,15,16,17,18], we decided to investigate whether Δ^9^-THC-induced histone modifications are restricted to adolescent treatment. To this aim, we performed the same protocol of injections (Figure 1B), as well as the same time-course study of histone modifications, in adult female rats exposed to Δ^9^-THC (Figure 3).

In the hippocampus, H3K14ac was significantly increased (67%) 2 h after adult Δ^9^-THC treatment, whereas no significant changes were observed later.

In the nucleus accumbens, a significant increase of H3K14ac levels (34%) was observed 24 h after the last Δ^9^-THC injection, whereas no changes were detected at the other time points.

In the amygdala, both H3K9me2 and H3K27me3 levels were significantly decreased (31% and 36%, respectively) 2 h after the end of Δ^9^-THC treatment, and then returned to control 24 h later. At this same interval of time, H3K9ac was significantly reduced (39%). Lastly, 48 h after the last Δ^9^-THC injection, H3K14ac levels were significantly decreased (28%).

As a whole, these data suggest that adult Δ^9^-THC exposure induced changes that are restricted to just one histone modification occurring within 24 h after the cessation of the treatment, except for the amygdala, where changes in histone modifications were present in the entire monitored time window.

### 2.3. A Global View of the Effects of Δ^9^-THC Exposure on Histone Modifications

**Figure 4:**
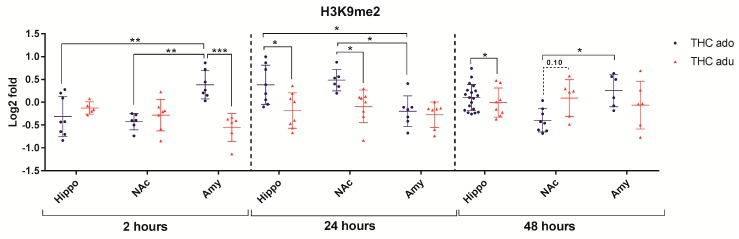
Comparison of Δ^9^-THC effect on H3K9me2 in adolescent (THC ado) and adult (THC adu) animals in the different brain areas. Data are reported as log2 fold. Mean ± SD are shown. * *p* < 0.05, ** *p* < 0.01, *** *p* < 0.001 (Tukey post-hoc test).

**Figure 5:**
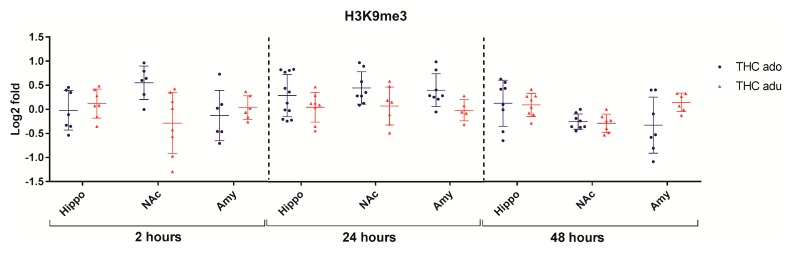
Comparison of Δ^9^-THC effect on H3K9me3 in adolescent (THC ado) and adult (THC adu) animals in the different brain areas. Data are reported as log2 fold. Mean ± SD are shown. Hippo, hippocampus; NAc, nucleus accumbens; Amy, amygdala.

**Figure 6:**
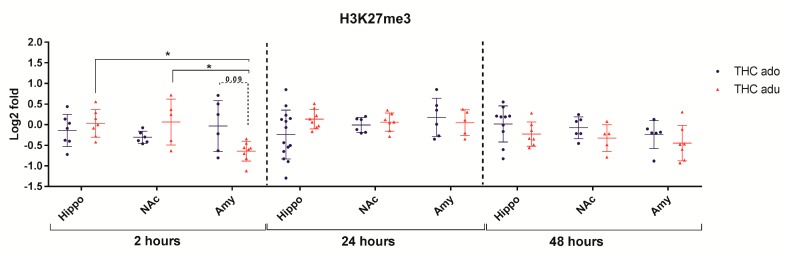
Comparison of Δ^9^-THC effect on H3K27me3 in adolescent (THC ado) and adult (THC adu) animals in the different brain areas. Data are reported as log2 fold. Mean ± SD are shown. * *p* < 0.05 (Tukey post-hoc test).

**Figure 7:**
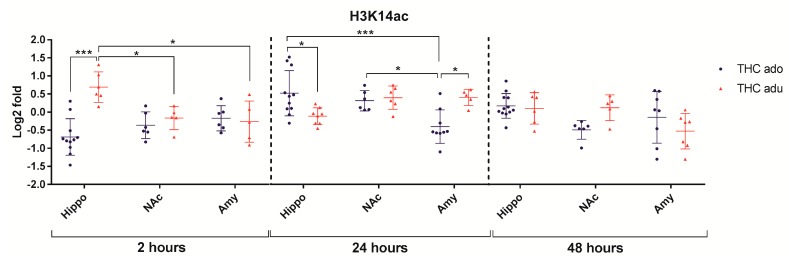
Comparison of Δ^9^-THC effect on H3K14ac in adolescent (THC ado) and adult (THC adu) animals in the different brain areas. Data are reported as log2 fold. Mean ± SD are shown. * *p* < 0.05, *** *p* < 0.001 (Tukey post-hoc test).

**Figure 8:**
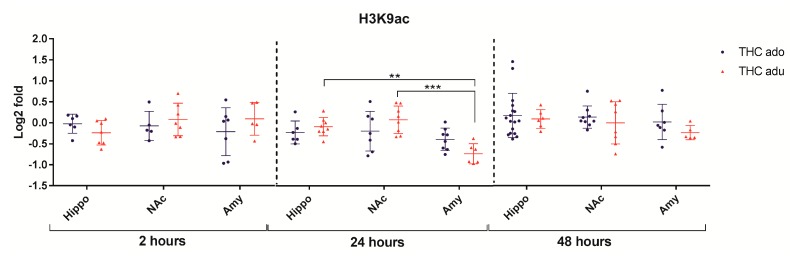
Comparison of Δ^9^-THC effect on H3K9ac in adolescent (THC ado) and adult (THC adu) animals in the different brain areas. Data are reported as log2 fold. Mean ± SD are shown. ** *p* < 0.01, *** *p* < 0.001 (Tukey post-hoc test).

In order to consider as a whole all the data retrieved from adolescent and adult rats exposed to Δ^9^-THC, we analyzed results in treated animals at each time point (i.e., 2, 24, and 48 h) by two-way multivariate analysis of variance (MANOVA), considering as independent variables the different brain areas and the age (adolescent vs. adult). Analysis of variance (ANOVA) and the Tukey post-hoc test were used to confirm the effects obtained via the MANOVAs and highlight significant (and relevant) contrasts. In almost all cases, the ANOVA confirmed the findings of the MANOVA. We decided to evidence relevant differences shown by the Tukey post-hoc test in Figure 4, Figure 5, Figure 6, Figure 7 and Figure 8. In particular, they highlight a different response between age groups in the same brain area, or among brain areas in the same age group.

Regarding H3K9me2 (Figure 4), the multivariate analysis revealed that: (i) age (*f* = 5.05; *p* = 0.035) and the interaction between the age and the brain area (*f* = 7.4; *p* = 0.0035) had significant main effects 2 h after the end of the treatment; (ii) age (*f* = 8.2; *p* = 0.0080) and the brain area (*f* = 6.8; *p* = 0.0040) had significant effects at 24 h; and (iii) the interaction between age and the brain area (*f* = 3.9; *p* = 0.031) had significant effects 48 h after the last injection.

Regarding H3K9me3 (Figure 5), the multivariate analysis revealed that age (*f* = 9.5; *p* = 0.0047) had significant effects at 24 h after the end of the treatment.

Regarding H3K27me3 (Figure 6), the multivariate analysis revealed that brain area (*f* = 4.03; *p* = 0.032) had significant main effects 2 h after the end of the treatment.

Regarding H3K14ac (Figure 7), the multivariate analysis revealed that: (i) age (*f* = 10.6; *p* = 0.0036) and the interaction between the age and the brain area (*f* = 5.8; *p* = 0.0093) had significant main effects 2 h after the end of the treatment; (ii) the interaction between the age and the brain area (F = 6.1; *p* = 0.0063) had significant main effects at 24 h.

Regarding H3K9ac (Figure 8), the multivariate analysis revealed that the brain area (*f* = 8.1; *p* = 0.0018) had significant main effects 24 h after the end of the treatment.

## 3. Discussion

The present study shows that chronic Δ^9^-THC exposure affects histone modifications in the brain of female rats in a region- and age-specific manner. Specifically, Δ^9^-THC acts on different targets depending on the considered brain area and, remarkably, the adolescent brain is generally more sensitive to Δ^9^-THC than the adult brain as evidenced by the multivariate analysis.

In the adolescent brain, from a kinetic point of view, we may describe two different epigenetic effects: one is mainly repressive, present immediately after the end of the treatment (2–24 h) and likely resulting directly from Δ^9^-THC exposure. The other, detectable in the next temporal window (i.e., from 24 h on), may represent a homeostatic response to counterbalance the Δ^9^-THC-induced repressive effect, since it has an opposite outcome at the transcriptional level. Specifically, the primary effect induced by adolescent Δ^9^-THC exposure is the negative modulation of gene transcription in all the analyzed brain areas, although this is caused by changes in different histone modifications. In the hippocampus, the primary effect is the reduction of H3K14ac, a marker known to promote gene expression, followed by a significant increase in di- and tri-methylation of H3K9, markers known to repress gene transcription, at 24 h. In the nucleus accumbens and the amygdala, the primary effect is represented by the increase of H3K9me, both H3K9me3 and H3K9me2, within 24 h of the end of the treatment. However, in the NAc, even the significant increase in H3K14 acetylation at 24 h could represent a primary event. Indeed, increased H3 acetylation in this brain area has been observed after chronic administration of different drugs of abuse [27,28,29], suggesting that it could be triggered by the activation of the mesolimbic reward circuitry.

These data join the results we have recently described in the prefrontal cortex [26], where the same adolescent Δ^9^-THC exposure in female rats increased methylation of H3K9 as a primary effect immediately after the treatment. Collectively, these data suggest that H3K9me may represent a common target affected by Δ^9^-THC in different brain areas, whereas the increased acetylation described in the NAc may be due to Δ^9^-THC activation of the reward circuitry.

This first wave of Δ^9^-THC-induced histone changes drives later chromatin rearrangements that, again, are area-specific. Indeed, in the hippocampus, we observed a switch from down- to upregulation of H3K14ac levels at 24 h. All the observed histone changes returned to control levels 48 h after the end of the treatment. In the nucleus accumbens, the enhancements in H3K9 methylation and H3K14 acetylation were completely reversed at 48 h, when a significant reduction was present. These further histone changes suggest the presence of a homeostatic response to the primary effect induced by Δ^9^-THC in the adolescent brain, as we have previously described also in the prefrontal cortex [26]. The only exception is represented by the amygdala, where primary histone modifications were reported to control levels without further mechanisms of counterbalance.

An important finding of this study regards the age-dependency of Δ^9^-THC-induced histone modifications. Indeed, adult female rats exposed to chronic Δ^9^-THC showed a different pattern of histone alterations: whilst the primary effect in the adolescent brain is represented by changes leading to transcriptional repression, the effect observed after adult treatment leads to transcriptional activation. Accordingly, a significant increase in H3K14 acetylation was present in the hippocampus and NAc, respectively, 2 and 24 h after the end of the adult treatment. Although under very different experimental conditions, Bilkei-Gorzo and colleagues have recently reported [30] similar opposing effects of Δ^9^-THC exposure in the hippocampus of young and mature mice. Indeed, they reported increased H3 acetylation after chronic Δ^9^-THC exposure in mature mice but a decreased trend in young animals, thus strengthening the hypothesis of an age-dependent effect of Δ^9^-THC on histone modifications. Regarding the increase in NAc H3K14 acetylation, this was also observed after adolescent treatment, strengthening the hypothesis that it may be linked to the activation of the mesolimbic rewarding circuitry [27,28,29]. Consistently, previous studies demonstrated that alterations in NAc histone acetylation affected addiction-related behaviors such as place conditioning and locomotor responses to psychostimulants [31].

Intriguingly, a more complex picture is present in the adult amygdala. The primary effect of Δ^9^-THC exposure in the amygdala is represented by a decrease in H3K9 di-methylation and H3K27 tri-methylation, which leads to transcriptional activation. In adolescent animals, we instead observed an increase in H3K9 methylation that returned to control levels within 48 h. This difference was further validated by the MANOVA analysis, which highlighted a significant association between H3K9 di-methylation and the adolescent/adult status 2 h after the end of the treatment. The univariate ANOVA, followed by the Tukey post-hoc test, confirmed this result and evidenced that the significance was due to opposite variations in the amygdala. Another peculiarity of the adult amygdala is the presence of a counterbalance event. Indeed, a second wave of changes that drive transcriptional repression, mediated by a decrease in H3 acetylation, is present 24 and 48 h after the end of the treatment. This suggests that the amygdala is more responsive in adult than adolescent animals. Again, these differences were evidenced by the MANOVA, with a significant effect of the interaction between the age and the brain area on H3K14ac. The univariate ANOVA confirmed the effect of the brain area and the post-hoc test highlighted the difference between adolescent amygdala and adult amygdala. Since it has been established that the amygdala is activated during exposure to aversive stimuli, functioning as a “behavioral brake” [32], the different response between adult and adolescent animals could represent the biological bases of the adolescent propensity for risk-taking and novelty-seeking behaviors. Remarkably, also in adolescent humans, neuroimaging studies have shown a weaker involvement of the amygdala, and a greater contribution of the NAc, in response to negative and positive stimuli compared to adults [33]. This fits well with the triadic model of neurodevelopment [34], suggesting that motivated behavior is mediated by the tension between reward (NAc) and threat (amygdala) systems. According to this model, adolescence would be characterized by an imbalance in the tension between reward and threat systems in favor of reward. This would explain the increase in reward seeking and lower regard for negative consequences that characterize adolescent behavior. In line with this model, our results showed a greater responsiveness of the adolescent NAc and a weaker sensitivity of the amygdala to Δ^9^-THC exposure compared to adults.

## 4. Materials and Methods

### 4.1. Drugs and Treatments

Δ^9^-THC was generously offered by GW Pharmaceuticals (Salisbury, UK). Δ^9^-THC was dissolved in ethanol, Kolliphor, and saline (1:1:18).

Adolescent (35–45 postnatal day, PND) and adult (75–85 PND) female Sprague-Dawley rats (Charles River, Calco, Italy) were treated with increasing intraperitoneal (ip) doses of Δ^9^-THC twice a day (2.5, 5, and 10 mg/kg) or its vehicle (Figure 1A,B), as previously described [13]. According to the transformation in human equivalent dose proposed by the Food and Drug Administration (FDA) and the average content of Δ^9^-THC in a joint, this protocol mimics a heavy cannabis abuse. Indeed, the first dose approximately corresponds to one joint containing 7% of Δ^9^-THC, the second one to two joints, and the higher one to four joints [35]. Currently, existing strains of cannabis have reached a content of Δ^9^-THC up to 14%. In this case, our treatment would mimic the consumption of half a joint (first dose), one joint (second dose), and two joints (third dose). All the experiments were carried out in strict accordance with the guidelines for care and use of experimental animals in the European Communities Council directive (2010/63/UE L 276, 20 October 2010) and approved by the Ethical Committee for Animal Research at the University of Insubria and by the Italian Ministry of Health (Aut. N.302/2015-PR). All efforts were made to reduce the suffering and the number of animals utilized.

### 4.2. Biochemical Assays

Brain areas (Hippo, NAc, and Amy) were obtained by regional dissection on ice, immediately frozen in liquid nitrogen, and stored at −80 °C until processing.

Histone extraction: Brain areas were homogenized in ice-cold buffer (50 mM Tris-HCl pH 7.5, 1 mM ethylenediaminetetraacetic acid (EDTA), 5 mM MgCl2, NaCl 50 mM, 5% glycerol, 1% Triton, 2 mM dithiothreitol (DTT), 2 mM phenylmethane sulfonyl fluoride (PMSF), 2 µg/mL aprotinin, 2 µg/mL leupeptin, 50 mM NaF) and centrifuged at 13,000 rpm at 4 °C for 5 min. The pellet was resuspended in nuclear lysis buffer (20 mM Hepes pH 8, 1.5 mM MgCl2, 420 mM NaCl, 2 mM DTT, 2 mM PMSF, 0.2 mM EDTA, 50 mM NaF, 25% glycerol, 10 μg/mL aprotinin, and 10 μg/mL leupeptin) and incubated on ice for 30 min with mild agitation. The samples were centrifuged at 13,000 rpm for 10 min at 4 °C. The pellet was resuspended in 0.2 M HCl, incubated on ice for 30 min with mild agitation and then overnight at 4 °C to acid extract histones. The samples were then centrifuged at 10,000 rpm for 10 min at 4 °C. The supernatant containing the histone proteins was mixed with six volumes of cold acetone and incubated at −20 °C overnight to precipitate histones. The day after, the samples were centrifuged at 4000 rpm at 4 °C for 10 min and the pellets were resuspended in radioimmunoprecipitation assay (RIPA) Buffer (50 mM Tris-HCl pH 7.5, 2 mM EDTA, NaCl 150 mM, Triton 1%, PMSF 2 mM, 5 µg/mL aprotinin, 5 µg/mL leupeptin). The protein concentration was determined according to the Micro-BCA assay kit (Pierce, Rockford, IL, USA).

Western Blot: Western blot was essentially performed as previously reported [26]. Briefly, proteins were resolved by 14% SDS-PAGE, blotted to polyvinylidene difluoride membrane, blocked for 2 h at room temperature in 5% dry skimmed milk in TBS-tween 20 (0.1%) before incubation overnight at 4 °C with the primary antibody. Primary antibodies were: (I) monoclonal anti-histone H3 di-methyl K9 (1:1000, AbCam, Cambridge, UK), (II) polyclonal anti-histone H3 tri-methyl K9 (1:1000, AbCam), (III) polyclonal anti-histone H3 tri-methyl K27 (1:1000, Merck Millipore, Darmstadt, Germany), (IV) polyclonal anti-histone H3 acetyl K9 (1:1000, Merck Millipore), monoclonal anti-histone H3 acetyl K14 (1:1000, Merck Millipore), (V) polyclonal anti-histone H3 (1:5000, AbCam). Bound antibodies were detected with horseradish peroxidase linked anti-rabbit or anti-mouse antibody (1:3000/5000, Chemicon International, Temecula, CA, USA). G:BOX Imaging System (Syngene, Cambridge, UK) was used for image acquisition. The relevant immunoreactive bands were quantified using ImageJ software. Representative blots are shown in Figure S1 (Supplementary Materials). Expression of the proteins was normalized to total histone H3. Data are presented as mean ± SD. Statistical analyses were performed using Student’s *t*-test for vehicle vs. Δ^9^-THC- treated animal comparisons. Data on Δ^9^-THC- treated animals were further analyzed by two-way MANOVA (using Wilks’s Λ statistics). In particular, fold change values were log2 transformed and then analyzed by two-way MANOVA, one for each time point (2, 24, 48 h), considering the brain area (hippocampus, nucleus accumbens, and amygdala) and the age (adolescent and adult) as independent variables. Results were confirmed by two-way ANOVA (one for each time point/histone modification pair), followed by the Tukey post-hoc test with the Benjamini-Hochberg correction of *p* values, in order to highlight significant contrasts.

## 5. Conclusions

Results shown in this paper suggest that Δ^9^-THC triggers a more complex response in the adolescent brain than in the adult brain. This response is characterized by a primary effect followed by compensatory changes (this paper and [26]), except in the amygdala. These changes may perturb adolescent brain refinement and, eventually, contribute to the behavioral alterations observed after adolescent Δ^9^-THC (see for review [36]). The presence of compensatory events may be part of the mechanisms that make the adolescent brain more vulnerable to Δ^9^-THC adverse effects.

## Supplementary Materials

Supplementary materials can be found at www.mdpi.com/1422-0067/18/10/2094/s1.
