# Supplementary material for: Chronic Δ9-THC Exposure Differently Affects Histone Modifications in the Adolescent and Adult Rat Brain
Source: Int J Mol Sci. 2017 Oct 4;18(10):2094. doi: 10.3390/ijms18102094 (PMC5666776; doi:10.3390/ijms18102094)
Supplement: Supplementary file 1 [file ijms-18-02094-s001.pdf]

Supplementary Materials

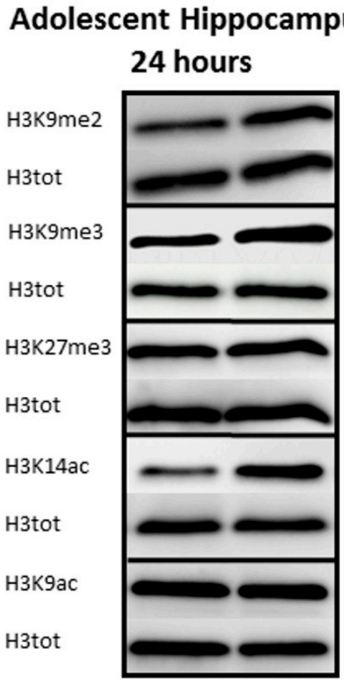

Figure S1. Representative blots of adolescent THC effect on histone modifications occurring in the hippocampus 24 hours after the last THC injection.
